# The risk of fibromyalgia in patients with iron deficiency anemia: a nationwide population-based cohort study

**DOI:** 10.1038/s41598-021-89842-9

**Published:** 2021-05-18

**Authors:** Wei-Cheng Yao, Hsuan-Ju Chen, Kam-Hang Leong, Kai-Lan Chang, Yu-Ting Tina Wang, Li-Chin Wu, Po-Ya Tung, Chien-Feng Kuo, Che-Chen Lin, Shin-Yi Tsai

**Affiliations:** 1 Department of Anesthesiology and Pain Medicine, Min-Sheng General Hospital, Taoyuan City, Taiwan; 2 College of Medicine, China Medical University, Taichung City, Taiwan; 3 Management Office for Health Data, China Medical University Hospital, Taichung City, Taiwan; 4 Department of Medicine, Mackay Medical College, New Taipei City, Taiwan; 5 Department of Laboratory Medicine, MacKay Memorial Hospital, Taipei City, Taiwan; 6 Division of Infectious Diseases, Department of Internal Medicine, Mackay Memorial Hospital, Taipei, Taiwan; 7 Department of Cosmetic Applications and Management, MacKay Junior College of Medicine, Nursing and Management, New Taipei City, Taiwan; 8 Healthcare Service Research Center (HSRC), Taichung Veterans General Hospital, Taichung, Taiwan; 9 Graduate Institute of Biomedical Sciences; Graduate Institute of Long-Term Care, Mackay Medical College, New Taipei City, Taiwan; 10 Department of Health Policy and Management, Johns Hopkins Bloomberg School of Public Health, Johns Hopkins University, Baltimore, MD USA

**Keywords:** Health care, Medical research, Risk factors

## Abstract

Since iron is essential for neurotransmitter synthesis, decreased iron stores might lead to reduced production of biogenic amines which phenomenon was shown in Fibromyalgia (FM) patients. The aims are to investigate the association of iron deficiency anemia (IDA) and FM and to find the effects of different interventions. We conducted a study using the Taiwan National Health Insurance Research Database. The IDA cohort consisted of 13,381 patients with newly diagnosed IDA between 2000 and 2008. Each patient with IDA was frequency-matched with one people without IDA, by sex, age and index year. The Cox proportional hazards regression analysis was conducted to estimate the association between IDA and FM risk. The event was the occurrence of FM. The overall incidence density rate of FM in the IDA cohort was higher than in the non-IDA cohort with a multivariable Cox proportional hazards model measured adjusted hazard ratio [HR], 1.19; 95% confidence interval [CI], 1.13–1.25). When using non-IDA group as reference, we compared with different therapies for IDA. The adjusted HRs of FM were 1.38 (95% CI = 1.30–1.47), 1.10 (95% CI = 1.03–1.16), 1.18 (95% CI = 0.98–1.43) and 0.73 (95% CI = 0.58–0.90) for IDA patient without therapy, iron supplement alone, blood transfusion alone and both iron supplement and blood transfusion respectively. Our results suggest IDA is associated with an increased risk of FM. All patients should have iron supplementation both to correct anemia and replenish body stores.

## Introduction

Iron deficiency anemia (IDA) is caused by reduced hemoglobin biosynthesis in erythrocytes due to iron deficiency^1^. According to iron deficiency staging guidelines, a serum ferritin level of < 60 mcg/dL is classified as deficiency with mild anemia, whereas a serum ferritin level of < 40 mcg/dL is classified as deficiency with severe anemia^1^. It is estimated that iron deficiency contributes to approximately 50% of all anemia cases in women^2^. In Taiwan, the prevalence of IDA is approximately 0.2% and 2.1% in male and female patients, respectively^3^. Fibromyalgia (FM) is a condition with an unknown etiology that is often characterized by chronic widespread musculoskeletal pain, fatigue, cognitive impairment, and psychiatric symptoms^4^. Although patients with non-anemic FM have relatively similar serum iron levels as compared with healthy individuals^5^, patients with IDA have a higher risk of developing FM compared to patients without IDA. This phenomenon may be explained through the pathophysiology of FM, as the pain experienced in FM patients are due to nervous system dysfunction caused by decreased levels of biogenic amine metabolites^6^. Because iron is a cofactor for several enzymes involved in the synthesis of neurotransmitters, such as tryptophan hydroxylase (for serotonin) and tyrosine hydroxylase (for norepinephrine and dopamine)^7^, decreased production of these biogenic amines in patients with FM may reflect iron deficiency. Furthermore, mean serum ferritin levels in patients with FM were found to be significantly lower than that in their healthy counterparts^8^. However, the association between IDA and FM has not been adequately investigated yet, and whether the management of IDA in patients affects the risk of developing FM requires further evaluation. This study aims to investigate the associations between IDA, FM, as well as discuss the different interventions for IDA.

## Materials and methods

Taiwan’s National Health Insurance (NHI) program is extensive as it covers 99% of Taiwanese citizens. The Longitudinal Health Insurance Database (LHID) contains the records of 1 million randomly sampled NHI insurants and is the representative of Taiwan’s NHI cohort. Specifically, the LHID documents the same health-related information as Taiwan’s NHI, including patients’ age, sex, occupation, and inpatient and outpatient records, and diagnoses are categorized according to the International Classification of Diseases, Ninth Revision, Clinical Modification (ICD-9-CM). According to previously established study designs^9–11^, we conducted this study using data from the LHID. The present study was an analysis of de-identified and encrypted secondary data; therefore, no informed consent was required. This study was approved by the Institutional Review Board of China Medical University (CMUH-104-REC2-115(CR-4)) and Mackay Memorial Hospital (16MMHIS074). We confirmed that all the methods in this research were performed in accordance with the relevant guidelines and regulations.

**Figure 1:**
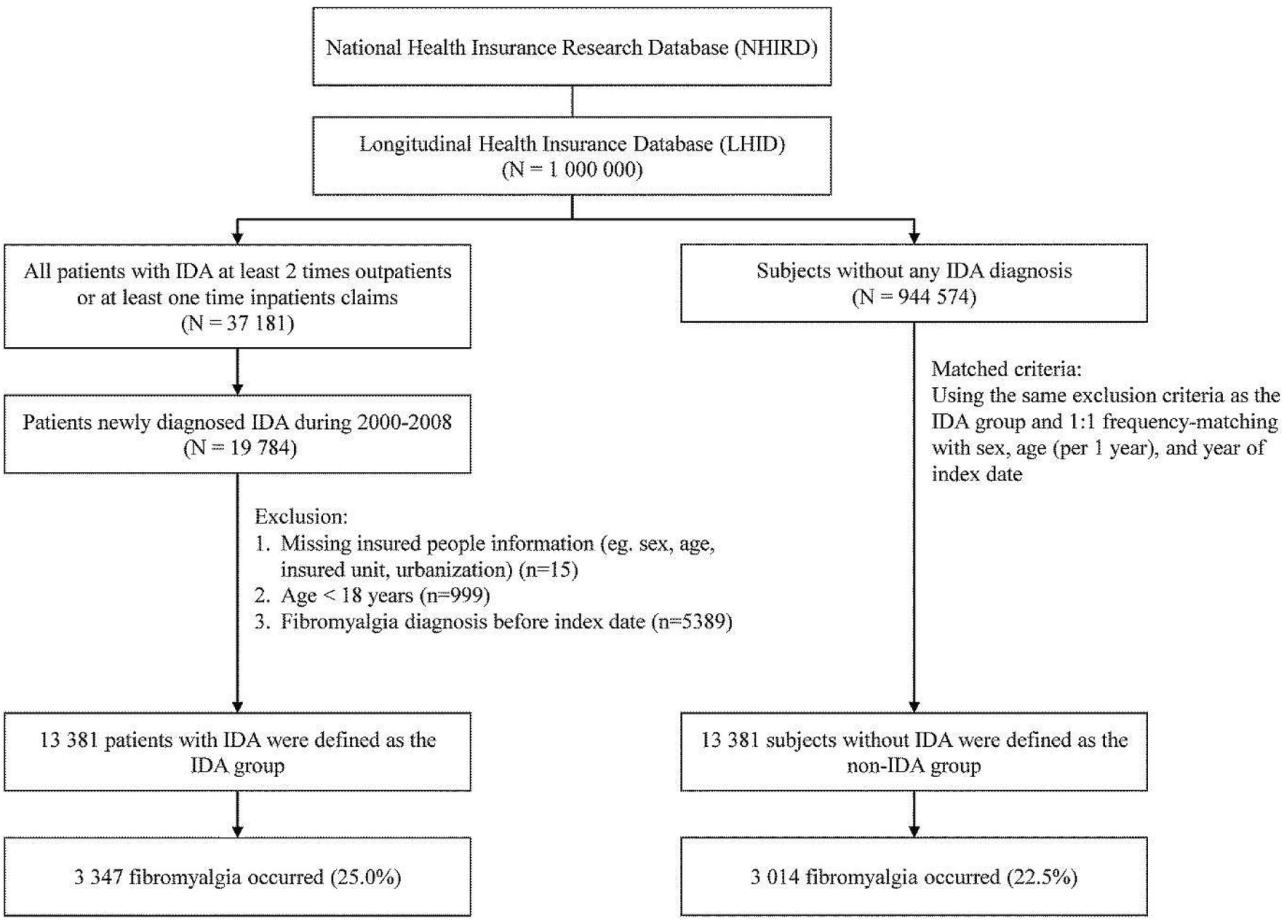
The selection process of the participants in the cohort study. Abbreviation: IDA, iron deficiency anemia.

We conducted a population-based retrospective cohort study to investigate the association between IDA and FM occurrence. Figure 1 illustrates the selection process. We constructed 2 separate cohorts, namely IDA and non-IDA cohorts. The IDA cohort consisted of patients newly diagnosed with IDA (ICD-9-CM 280.xx) between the years 2000 and 2008 in patients aged ≥ 18 years. The date of diagnosis was considered as the index date. Equal numbers of age- and sex-matched healthy participants were selected to construct the matched non-IDA cohort. The index date for the non-IDA participants was randomly generated using the same index year as that for the matched participants with IDA. Participants with a history of FM (ICD-9-CM 729.1x) before the index date were excluded. We also considered the effects of the type of IDA therapy and classified the treatment types into 4 subgroups, namely: (i) without therapy, (ii) iron supplementation alone, (iii) blood transfusion alone, and (iv) a combination of iron supplementation and blood transfusion. The study participants were followed up from the index date. We set the endpoint of the study at development of FM, based on the ICD-9 codes (ICD-9-CM 729.1x).

Baseline comorbidities referred to those diagnosed before the index date. These included diabetes (ICD-9-CM 250.xx), obesity (ICD-9-CM 278.0x), osteoarthritis (ICD-9-CM 715.xx), sleep disorders (ICD-9-CM 307.xx, 327.xx, and 780.5x), irritable bowel syndrome (IBS; ICD-9-CM 564.1x), migraine (ICD-9-CM 346.xx), temporomandibular joint (TMJ) disorders (ICD-9-CM 524.6x), depression (ICD-9-CM: 296.2x, 296.3x, 300.4x, and 311.xx), and anxiety (ICD-9-CM 300.00).

Regarding the descriptive statistics of the IDA and non-IDA cohorts, continuous variables were summarized as means and standard deviations, and categorical variables were presented as the number of cases and percentages. Chi-square and t-tests were used to analyze the distribution differences between the IDA and non-IDA cohorts. The cumulative incidence curve was evaluated using the Kaplan–Meier method, and the curve difference was assessed using the log-rank test. To determine the risk of FM among IDA and non-IDA cohorts, we applied single variable and multiple Cox proportional hazard models to estimate the hazard ratios (HRs) and corresponding 95% confidence intervals (CIs). All statistical analyses were conducted using the SAS package (version 9.4; SAS Institute Inc., Cary, NC, United States). The incidence curve was generated using R software (R Foundation for Statistical Computing, Vienna, Austria). A two-tailed p value less than 0.05 was considered statistically significant.

## Results

As shown in Table 1, the mean age of the IDA and non-IDA cohorts was 50.3 years. For both cohorts, 25.8% were men. With the exemption of the comorbidities obesity and TMJ disorders, overall the prevalence of comorbidities was higher in the IDA cohort than compared to the non-IDA cohort (*p* < 0.001).

**Table 1:** Demographic factors and comorbidity of study participants according to iron deficiency anemia status.

Variable	IDA group N = 13,381		Non-IDA group N = 13,381		*p* value
	n	%	n	%	
Sex					> 0.99
Women	9927	74.2	9927	74.2	
Men	3454	25.8	3454	25.8	
Age, years					> 0.99
18–39	4210	31.5	4210	31.5	
40–59	5096	38.1	5096	38.1	
≥ 60	4075	30.5	4075	30.5	
Means (SD)^†^	50.3	(18.7)	50.3	(18.7)	0.95
Comorbidity					
Diabetes	1724	12.9	1082	12.9	< 0.001
Obesity	87	0.65	73	0.55	0.30
Osteoarthrosis	2491	18.6	1975	14.8	< 0.001
Sleep disorder	2918	21.8	1946	14.5	< 0.001
Irritable bowel syndrome	954	7.13	531	3.97	< 0.001
Migraine	454	3.39	318	2.38	< 0.001
TMJ	86	0.64	64	0.48	0.09
Depression	754	5.63	448	3.35	< 0.001
Anxiety	972	7.26	632	4.72	< 0.001

*IDA* iron deficiency anemia, *SD* standard deviation.

^†^Student’s t-test.

**Figure 2:**
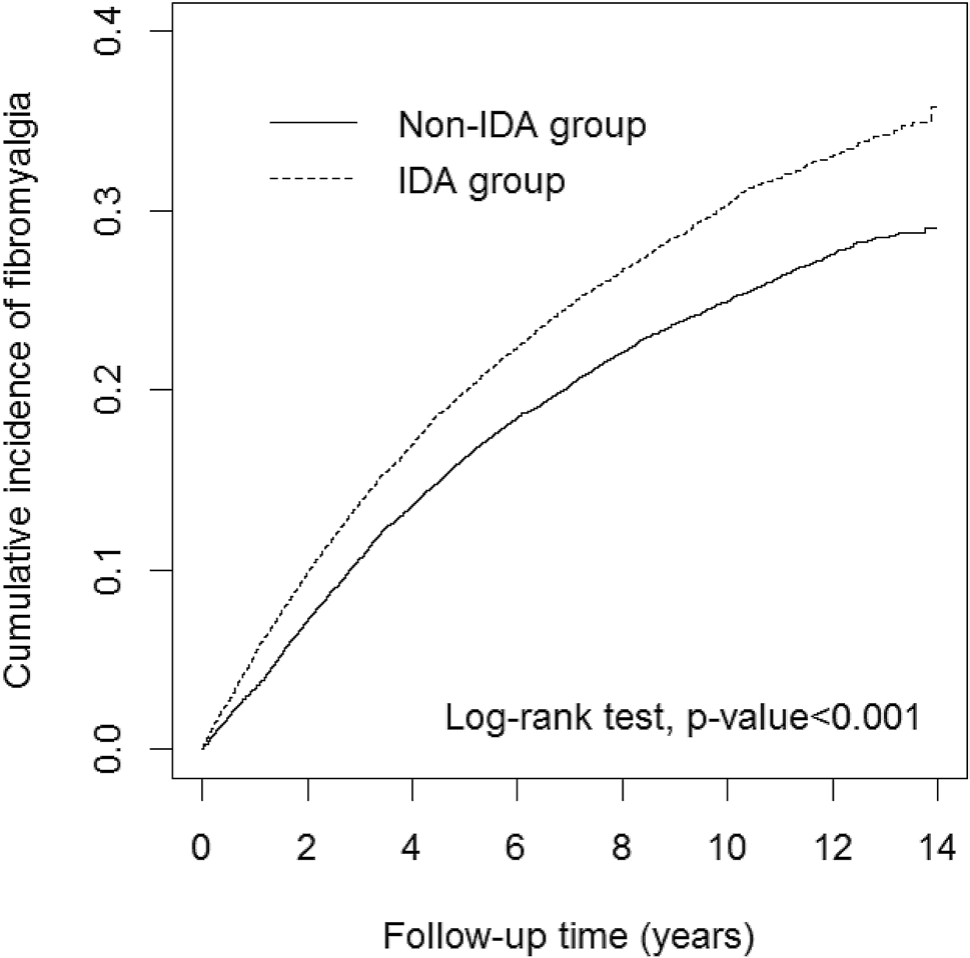
Cumulative incidence curves of fibromyalgia for groups with and without iron deficiency anemia. Abbreviation: IDA, iron deficiency anemia.

Figure 2 presents FM incidence curves for the two cohorts. We found that the IDA cohort had a significantly higher FM incidence than the non-IDA cohort (log-rank *p* < 0.001). Table 2 shows the incidence and risk of FM between the IDA and non-IDA cohorts. The incidence density of FM was 29.5 and 37.8 per 1000 person-years for the IDA and non-IDA cohorts, respectively. Patients with IDA also had a significantly greater risk of developing FM than their matching counterparts (HR, 1.19; 95% CI, 1.13–1.25). In the full model that included sex, age, patients with IDA, and comorbidities, we revealed that female patients (HR, 1.24; 95% IC, 1.16–1.32) and patients with obesity (HR, 1.42; 95% CI, 1.09–1.85), osteoarthritis (HR, 1.51; 95% CI, 1.41–1.62), sleep disorders (HR, 1.36; 95% CI, 1.27–1.44), IBS (HR, 1.25; 95% Cl, 1.13–1.38), migraine (HR, 1.33; 95% CI, 1.18–1.51), and anxiety (HR, 1.19; 95% CI, 1.08–1.31) were associated with higher risks of developing FM.

**Table 2:** Cox model measured hazard ratios and 95% confidence interval of fibromyalgia associated with iron deficiency anemia and covariates.

Variable	Event no	Person-years	Incidence density^#^	HR (95%CI)	
				Unadjusted	Adjusted^†^
**Iron deficiency anemia**					
No	3014	102,479	29.5	Ref	Ref
Yes	3347	88,641	37.8	1.26 (1.20–1.32)	1.19 (1.13–1.25)
**Sex**					
Women	5129	147,980	34.7	1.24 (1.16–1.32)	1.24 (1.16–1.32)
Men	1245	43,140	28.9	Ref	Ref
**Age, years**					
18–39	1877	70,076	26.8	Ref	Ref
40–59	2907	76,051	38.2	1.40 (1.32–1.48)	1.32 (1.24–1.40)
≥ 60	1590	44,992	35.3	1.23 (1.15–1.32)	1.05 (0.97–1.14)
**Comorbidity**					
**Diabetes**					
No	5777	175,900	32.8	Ref	Ref
Yes	597	15,220	39.2	1.13 (1.04–1.23)	0.97 (0.89–1.06)
**Obesity**					
No	6319	190,127	33.2	Ref	Ref
Yes	55	994	55.4	1.59 (1.22–2.08)	1.42 (1.09–1.85)
**Osteoarthrosis**					
No	5061	166,388	30.4	Ref	Ref
Yes	1313	24,732	53.1	1.65 (1.55–1.75)	1.51 (1.41–1.62)
**Sleep disorder**					
No	4919	162,811	30.2	Ref	Ref
Yes	1455	28,309	51.4	1.61 (1.52–1.71)	1.36 (1.27–1.44)
**Irritable bowel syndrome**					
No	5928	182,358	32.5	Ref	Ref
Yes	446	8762	50.9	1.50 (1.37–1.66)	1.25 (1.13–1.38)
**Migraine**					
No	6100	186,279	32.8	Ref	Ref
Yes	274	4841	56.6	1.67 (1.48–1.89)	1.33 (1.18–1.51)
**TMJ**					
No	6330	190,150	33.3	Ref	Ref
Yes	44	970	45.4	1.32 (0.98–1.77)	1.08 (0.80–1.46)
**Depression**					
No	6025	184,138	32.7	Ref	Ref
Yes	349	6982	50.0	1.46 (1.31–1.63)	1.08 (0.96–1.21)
**Anxiety**					
No	5864	181,973	32.2	Ref	Ref
Yes	510	9147	55.8	1.64 (1.49–1.79)	1.19 (1.08–1.31)

*HR* hazard ratio, *CI* confidence interval.

^#^Per 1000 person-years.

^†^Multivariate Cox proportional hazards regression model including iron deficiency anemia sex, age, diabetes, obesity, osteoarthrosis, sleep disorder, irritable bowel syndrome, migraine, TMJ, depression, and anxiety.

Table 3 summarizes the risk of FM stratified by sex, age, and comorbidity. Women with IDA had a 1.22-fold higher risk of developing FM compared with individuals without IDA (HR, 1.22; 95% CI, 1.15–1.29). However, the risks of developing FM were similar between men with and without IDA (HR, 1.06; 95% CI, 0.94–1.18). Our results showed that the HRs of FM for patients with IDA aged 18–39 years, 40–59 years, and ≥ 60 years were 1.34 (95% CI, 1.22–1.47), 1.15 (95% CI, 1.07–1.24), and 1.08 (95% CI, 0.98–1.19), respectively. IDA patients without comorbidities had a 1.22-fold higher risk of developing FM over their patients without IDA and comorbidities, whereas patients with IDA and comorbidities had a 1.15-fold higher risk of developing FM over their non-IDA counterparts with comorbidities.

**Table 3:** Incidence density and hazard ratios of fibromyalgia according to iron deficiency anemia status stratified by sex, age, and comorbidity.

Variables	IDA group			Non-IDA group			HR (95% CI)	
	Event no	Person-year	Incidence density^#^	Event no	Person-year	Incidence density^#^	Crude	Adjusted^†^
**Sex**								
Women	2761	69,994	39.5	2368	77,986	30.4	1.28 (1.21–1.36)	1.22 (1.15–1.29)
Men	586	18,647	31.4	659	24,493	26.9	1.14 (1.02–1.27)	1.06 (0.94–1.18)
**Age, years**								
18–39	1079	33,962	31.8	798	36,114	22.1	1.43 (1.30–1.56)	1.34 (1.22–1.47)
40–59	1531	35,731	42.9	1376	40,320	34.1	1.24 (1.15–1.33)	1.15 (1.07–1.24)
≥ 60	737	18,948	38.9	853	26,045	32.8	1.15 (1.04–1.27)	1.08 (0.98–1.19)
**Comorbidity status**^‡^								
No	1586	53,571	29.6	1769	72,980	24.2	1.22 (1.14–1.30)	1.22 (1.14–1.30)
Yes	1761	35,070	50.2	1258	29,499	42.7	1.16 (1.08–1.25)	1.15 (1.07–1.24)

*IDA* iron deficiency anemia, *HR* hazard ratio, *CI* confidence interval.

^#^Per 1000 person-years.

^†^Model mutually adjusted for sex, age, and each comorbidity (including diabetes, obesity, osteoarthrosis, sleep disorder, irritable bowel syndrome, migraine, TMJ, depression, and anxiety).

^‡^Patients with any one of diabetes, obesity, osteoarthrosis, sleep disorder, irritable bowel syndrome, migraine, TMJ, depression, and anxiety were classified as the comorbidity group.

Table 4 shows the risks of FM in patients receiving various IDA treatments. Compared with the non-IDA group, the HRs of FM for patients with IDA who received the following treatments were as follows: no treatment, HR of 1.38 (95% CI, 1.30–1.47); blood transfusion only, 1.18 (95% CI, 0.98–1.43); iron supplementation only, 1.10 (95% CI, 1.03–1.16); and lastly, combination of iron supplementation and blood transfusion, 0.73 (95% CI, 0.58–0.90). We also compared the HRs for different IDA therapies using patients with IDA who received no therapy as a reference. The adjusted HRs of FM for patients with IDA who received blood transfusion only, iron supplementation only, and combination of iron supplementation and blood transfusion were 0.88 (95% CI 0.73–1.07), 0.79 (95% CI, 0.73–0.85), and 0.54 (95% CI, 0.43–0.67), respectively.

**Table 4:** Incidence density and hazard ratios of fibromyalgia for iron deficiency anemia status combined with therapy (including iron supplementation and blood transfusion).

Variables	N	Event no	Person-year	Incidence density#	HR (95% CI)			
					Crude	Adjusted^†^	Crude	Adjusted^†^
Non-IDA group	13,381	3027	102,479	29.5	Ref	Ref		
**IDA group**								
Without therapy	5355	1445	33,119	43.6	1.45 (1.36–1.55)	1.38 (1.30–1.47)	Ref	Ref
Blood transfusion alone	694	116	3097	37.5	1.20 (0.99–1.44)	1.18 (0.98–1.43)	0.82 (0.68–0.99)	0.88 (0.73–1.07)
Iron supplementation alone	6777	1703	49,022	34.7	1.17 (1.10–1.24)	1.10 (1.03–1.16)	0.81 (0.75–0.87)	0.79 (0.73–0.85)
Combination of iron supplementation and blood transfusion	555	83	3404	24.4	0.80 (0.64–0.99)	0.73 (0.58–0.90)	0.55 (0.44–0.68)	0.54 (0.43–0.67)

*IDA* iron deficiency anemia, *HR* hazard ratio, *CI* confidence interval.

^#^Per 1000 person-years.

^†^Model adjusted for sex, age, and each comorbidity (including diabetes, obesity, osteoarthritis, sleep disorder, irritable bowel syndrome, migraine, TMJ, depression, and anxiety).

## Discussion

Our results suggest that the overall incidence of FM was higher in patients with IDA than in the non-IDA controls. Moreover, IDA patients who were women, aged between 18 and 59 years, and those with IDA either with or without comorbidities had significantly increased hazard ratios (HRs) for FM. We also revealed that patients with IDA who received the combination therapy of iron supplementation and blood transfusion had a lower HR of FM than patients with IDA who received no therapy. Considering the treatment effects and the risk of FM within our IDA cohort, patients who received iron supplementation generally had significantly lower HRs for FM than those who received blood transfusion alone.

Our findings were consistent with those of previous studies suggesting that women with IDA had a higher risk of developing FM^8,12^. The difference in FM prevalence between men and women may be due to a variety of factors, including the natural course of FM, the effect of diagnostic criteria, and the willingness to seek health care^13^. In the general population, the prevalence and incidence of FM increases with age, especially with the peak prevalence at middle age (30–50 years) and older age (50–70 years) groups^13–15^. However, we observed a higher risk of FM in patients with IDA aged 18 to 59 years. Compared with non-IDA controls, the risk of FM was higher in those with IDA regardless of comorbidities, strongly suggesting that iron homeostasis plays a crucial role in the development of FM.

A link between iron deficiency and FM has been postulated in many studies. Lower levels of iron, calcium, and magnesium were found in hair samples of women diagnosed with FM, compared with healthy controls^16^. Moreover, a study revealed that patients with FM had significantly lower mean serum ferritin levels than healthy controls (27 mg/mL ± 21 mg/mL vs. 44 mg/mL ± 31 mg/mL; *p* = 0.003). Similarly, a serum ferritin level of < 0.05 µg/mL was associated with a 6.5-fold increased risk of FM (*p* = 0.002)^8^. Our results suggest that the prevalence of FM is higher in the IDA population compared with those without IDA. Furthermore, we revealed that a decreased iron level is associated with an increased risk of FM, which could potentially be reversed by iron supplementation^8,12,17^.

In other relevant studies, mice with iron deficiency were found to have a lower pain threshold, similar to the proposed FM pathophysiology in humans^4,18^. According to the etiopathogenesis of FM in a recent review^19^, there are various complex and intricate molecular mediators involved, including: biogenic amine signaling, brain-derived neurotropic factor, inflammatory mediators, the hypothalamic–pituitary–adrenal axis within the sympathetic nervous system, oxidative stress, sex hormones and opioid peptides. Thus, a dysfunction of the descending pain inhibitory pathway due to abnormal neurotransmitter activities (such as depleted biogenic amine levels) may in turn contribute to the symptoms of FM^20^.

It has been reported that patients with FM have a lower concentration of tryptophan (a precursor of serotonin) and serotonin in plasma and thus overall have lower levels of serotonin, norepinephrine and dopamine metabolites in their cerebrospinal fluid (CSF) than healthy controls^21–23^. Iron is a cofactor for several enzymes responsible for the synthesis of these neurotransmitters, including tryptophan hydroxylase (for synthesizing serotonin) and tyrosine hydroxylase (for synthesizing norepinephrine and dopamine)^7^. Thus, a deficiency in iron may very well cause a cascade of effects in theory, such as the underutilization of tryptophan^24^, leading to lowering serotonin levels in the CSF that may explain the wide range of clinical symptoms manifested in FM patients.

Even though iron is an essential element for enzymes to synthesize certain neurotransmitters^25,26^, iron deficiency in the brain does not affect the enzymes involved in neurotransmitter synthesis in rats, indicating that more than one mechanism exists^27^. However, a phenomenon worth noting suggests that there is a lower density of serotonin-specific transporters in both iron-deficient rats and patients with FM, compared with cases without FM^25,28^. This means that synaptic vesicles in the brains of iron-deficient rats had poorer uptake of serotonin compared with control rats^29^. The iron deficiency may also decrease the number of D2 receptors and disrupt the reuptake of dopamine^30^, which many explain why metabolic defects and abnormal dopamine response were indicated in patients with FM^31,32^.

The 5-HT systems acting on the spinal cord can influence sensory, affective and autonomic components of pain^33^. The activation of the inhibitory dopamine D2 family receptors is associated with the analgesic mechanism of dopamine, a natural analgesia of the CNS system^34^. Thus, the process in which serotonin and dopamine are involved in the descending inhibition of pain as described above could explain why iron supplementation can reduce the risk of developing FM in iron deficient patients.

In patients with FM, iron deficiency may also decrease oxygen transport and mitochondrial function in muscles. This may affect exercise performance and cause frequent fatigue, one of the most prominent clinical symptoms of FM^35^.

In line with recent findings^17^, our results suggested that iron supplementation reduces the risk of FM. However, serum hemoglobin and iron levels were similar between patients with FM with or without IDA^5,12^. This may be explained in part that anemia often causes similar symptoms as FM, such as fatigue and cognitive dysfunction^36^. Compared with the control (IDA cohort that did not receive treatment), the HRs for FM were different in patients with IDA who received iron supplementation alone and iron supplementation in combination with blood transfusion, but not in patients who received blood transfusion alone, indicating that anemia cannot completely explain the development of FM.

Blood transfusions are not the recommended first line treatment for IDA—rather it is often used for the acute correction of hemoglobin levels in hemodynamically unstable patients in an emergent setting. Furthermore, blood transfusions do not correct nor improve the overall iron-deficiency circumstances and outcomes^37,38^.

Although the mechanism is not yet fully understood, iron supplementation reduces fatigue in individuals with non-anemic iron deficiency and may even increase productivity of those with IDA^39^. In agreement with previous findings, our results highlighted the importance of the correction of iron deficiency status.

As a high percentage of the population is enrolled in Taiwan’s NHI, the selection bias in this study was minimized. All data relating to diagnosis of diseases were coded using ICD-9 codes, which were established by clinicians and reviewed extensively by specialists for insurance claim purposes. Thus, the diagnostic history of patients in this database is highly reliable. However, there are not without limitations in this study. First, the ICD-9 used to define IDA and FM diagnoses did not document the disease severity of patients. To overcome this shortcoming, we utilized the frequency of blood transfusion to estimate and stratify the severity of IDA. Besides our proposed approach, other clinical evaluations such as serum hemoglobin and other biomarkers for IDA can be considered to see how IDA severity impacts FM development. Secondly, the study cohort only consisted of East Asians living in Taiwan, so our conclusions could be affected by geographical and ethnic differences as well as lifestyle, living conditions, among other factors not included in the database. Thus, further research is required to clarify this.

## Conclusions

In conclusion, we revealed that IDA increased the risk of developing FM, especially in women population, and proposed several possible mechanisms regarding the nature of FM pathophysiology which may provide insights for the further studies. In terms of therapy, iron supplementation may reduce IDA patients’ risk of developing FM. Furthermore, additional studies are required to evaluate how differences in IDA severity can affect the risk of developing FM.
