# Publisher Correction: The risk of fibromyalgia in patients with iron deficiency anemia: a nationwide population‑based cohort study

**DOI:** 10.1038/s41598-021-94527-4

**Published:** 2021-07-27

**Authors:** Wei-Cheng Yao, Hsuan-Ju Chen, Kam-Hang Leong, Kai-Lan Chang, Yu-Ting Tina Wang, Li-Chin Wu, Po-Ya Tung, Chien-Feng Kuo, Che-Chen Lin, Shin-Yi Tsai

**Affiliations:** 1 Department of Anesthesiology and Pain Medicine, Min-Sheng General Hospital, Taoyuan City, Taiwan; 2 College of Medicine, China Medical University, Taichung City, Taiwan; 3 Management Office for Health Data, China Medical University Hospital, Taichung City, Taiwan; 4 Department of Medicine, Mackay Medical College, New Taipei City, Taiwan; 5 Department of Laboratory Medicine, MacKay Memorial Hospital, Taipei City, Taiwan; 6 Division of Infectious Diseases, Department of Internal Medicine, Mackay Memorial Hospital, Taipei, Taiwan; 7 Department of Cosmetic Applications and Management, MacKay Junior College of Medicine, Nursing and Management, New Taipei City, Taiwan; 8 Healthcare Service Research Center (HSRC), Taichung Veterans General Hospital, Taichung, Taiwan; 9 Graduate Institute of Biomedical Sciences; Graduate Institute of Long-Term Care, Mackay Medical College, New Taipei City, Taiwan; 10 Department of Health Policy and Management, Johns Hopkins Bloomberg School of Public Health, Johns Hopkins University, Baltimore, MD USA

Correction to: *Scientific Reports* 10.1038/s41598-021-89842-9, published online 18 May 2021

The original version of this Article contained errors in the Affiliations.

Affiliation 5 was incorrectly given as ‘Department of Laboratory Medicine, Mackay Medical College, Taipei City, Taiwan’. The correct affiliation is listed below:

Department of Laboratory Medicine, MacKay Memorial Hospital, Taipei City, Taiwan.

Shin-Yi Tsai was incorrectly affiliated with ‘Healthcare Service Research Center (HSRC), Taichung Veterans General Hospital, Taichung, Taiwan’. The correct affiliations are listed below:

Affiliation 4 Department of Medicine, Mackay Medical College, New Taipei City, Taiwan.

Affiliation 5 Department of Laboratory Medicine, MacKay Memorial Hospital, Taipei City, Taiwan.

Affiliation 9 Graduate Institute of Biomedical Sciences; Graduate Institute of Long‑Term Care, Mackay Medical College, New Taipei City, Taiwan.

Affiliation 10 Department of Health Policy and Management, Johns Hopkins Bloomberg School of Public Health, Johns Hopkins University, Baltimore, MD, USA.

The affiliation for the author Chien-Feng Kuo was incomplete. The correct affiliations are listed below:

Affiliation 6 Division of Infectious Diseases, Department of Internal Medicine, Mackay Memorial Hospital, Taipei, Taiwan.

Affiliation 7 Department of Cosmetic Applications and Management, MacKay Junior College of Medicine, Nursing and Management, New Taipei City, Taiwan.

Finally, Che-Chen Lin was incorrectly affiliated with ‘Department of Cosmetic Applications and Management, MacKay Junior College of Medicine, Nursing and Management, New Taipei City, Taiwan’. The correct affiliation is listed below:

Affiliation 8 Healthcare Service Research Center (HSRC), Taichung Veterans General Hospital, Taichung, Taiwan.

The original Article has been corrected.

